# Prevalence of metabolic syndrome in patients with minor beta thalassemia and its related factors: a cross-sectional study

**DOI:** 10.1186/s40200-014-0108-z

**Published:** 2014-12-31

**Authors:** Mohammad Hossein Gozashti, Ali Hasanzadeh, Mahdieh Mashrouteh

**Affiliations:** Endocrinology, Physiology Research Center, Kerman University of Medical Sciences, Kerman, Iran; Kerman University of Medical Sciences, Kerman, Iran; Research Center for Modeling in Health, Institute for Future Studies in Health, Kerman University of Medical Sciences, Kerman, Iran

**Keywords:** Metabolic syndrome, Minor thalassemia, Prevalence

## Abstract

**Background:**

Atherosclerotic disorders, hypertension and lipid profile alterations are of a lower prevalence in patients with minor beta thalassemia. On the other hand, nowadays, metabolic syndrome is considered as one of the major risk factors of developing cardiovascular diseases. Therefore, the present study was performed to determine the prevalence of metabolic syndrome in patients with minor beta thalassemia.

**Methods:**

In this case-control study, body length, weight and waist circumference, blood pressure, fasting blood sugar [FBS], triglyceride, cholesterol, HDL, and LDL levels were determined in 150 patients with minor beta thalassemia and 300 healthy individuals as control group [matched based on age and sex]. The prevalence of metabolic syndrome was calculated based on ATPIII criteria. Data were analyzed through SPSS16 software package.

**Results:**

The prevalence of metabolic syndrome was 12.7% in the thalassemia group and 36.7% in the control group [p < 0.0001]. In the patient group, 3 ones [8.3%] of those with metabolic syndrome were male and 16 ones [14%] were female [p = 0.5]. Mean age of patients with metabolic syndrome was 39.4 ± 8.5 years and mean age of those without metabolic syndrome was 36.4 ± 7.8 years [p = 0.1]. Mean BMI of those with metabolic syndrome was 31.3 ± 4.1 kg/m^2^ and that of those without metabolic syndrome was 24.2 ± 4.4 kg/m^2^ [p < 0.0001].

**Conclusions:**

The obtained results show lower prevalence of metabolic syndrome in patients with minor thalassemia. Moreover, the prevalence of metabolic syndrome in patients with minor thalassemia showed no relationship with sex and age and these patients had just higher BMI.

## Introduction

Since 1960s, a relationship has been found between cardiovascular diseases and high level of serum triglyceride, severe obesity, insulin resistance, glucose intolerance and hypertension [1-4]. In 1988, for the first time, Reavan suggested the term of metabolic syndrome for combination of these cardiovascular factors [5]. Since insulin resistance has been considered as an underlying cause of risk factors and consequently development of diabetes and cardiovascular diseases [5-10], insulin resistance syndrome was used as another term for metabolic syndrome [11]. Genetic and racial factors and life style have role in metabolic syndrome [5-10].

Although there are several criteria for the diagnosis of metabolic syndrome, the most applicable one is clinical diagnosis based on specified criteria of ATPIII [Adult Treatment Panel III] and IDF [International Diabetes Federation] [5,6].

Today, metabolic syndrome is considered as one of the important problems of both developed and developing countries and its prevalence increases with urbanization increase [1-4]. The prevalence of metabolic syndrome has been reported 33.7% in a study on blood sugar and lipid levels of Tehran citizens [11].

In a study performed on rural and urban populations of Isfahan and Arak in 2006, the prevalence of metabolic syndrome, based on ATPIII criteria, has been reported approximately 21.9% in Iranian adults of central districts [12]. The prevalence of metabolic syndrome, based on a study performed on 984 citizens of Babol on 2009, was 31% [13].

Thalassemia is a heterogenic group of inherited anemia occurs due to the mutation in the gene related to the synthesis of hemoglobin beta- or alpha- chains. Patients with minor beta thalassemia have usually no problem clinically, but show a type of microcytic hypochromic anemia [14].

In a study performed on minor beta thalassemia carriers in Sardinia, not only LDL (low density of lipoprotein) level of thalassemia patients was lower compared to the control group, but also they showed significant decrease of Apo A and Apo B. Indeed, some alterations in LDL and HDL protein content have been reported and the researchers have suggested that in minor beta thalassemia patients, myocardial infarction happens 10 years later in comparison to the control group [15]. In Crowley et al. study [1987] on patients with myocardial infarction, the frequency of minor beta thalassemia was significantly lower and this disease had acted as a protective factor [16].

In Gallerani et al. study [1991], a negative relationship between acute myocardial infarction and minor beta thalassemia was observed only in male patients [17]. According to Wang and Schilling study [1995], the prevalence of minor beta thalassemia in patients with myocardial infarction is significantly lower than the control group [18]. Another study has also showed lower levels of total cholesterol and LDL in minor beta thalassemia patients [19].

Several studies have shown that hypertension and blood lipid disorders are less frequent in persons with minor beta thalassemia as compared to the normal population. On the other hand, nowadays, metabolic syndrome is considered as one of the major risk factors of developing cardiovascular diseases and diabetes. In the case of lower prevalence of metabolic syndrome in minor beta thalassemia patients, further studies can be performed in order to determine the cause of this fact and consequently causes of developing this syndrome.

Since the prevalence of metabolic syndrome in minor beta thalassemia patients have not been determined so far, we attempted to compare minor beta thalassemia patients with normal population in regard to the prevalence of metabolic syndrome.

## Method

This study was a case-control study. Our patients included 150 minor beta thalassemia patients. Subjects were parents of major beta thalassemia patients, aged ≥ 20 years and were randomly selected from the files in Samen-ol-hojaj Center for Special Diseases in Kerman/ Iran. In the case of patient refusal to participate in the study, another patient was substituted. A total of 300 healthy volunteered individuals matched for age and sex were selected as control group. They are selected from patients’ relatives or the center personnel. All subjects were ensured of the confidentiality of their information and were enrolled into the study after obtaining their written informed consent by center staffs. This study was approved by Kerman University of Medical Science.

After 12 hours fasting, FBS, triglyceride, cholesterol, HDL and LDL levels were determined by enzymatic methods. Body length and weight, waist circumference and blood pressure of subjects were recorded.

Weight was measured with the subject in least clothing and stocking feet [without shoes] by a digital scale with 100 g precision. Body length was measured by a tape while the subject was in standing position and stocking feet and shoulders were in normal position. Waist circumference was measured at narrowest point and at the end of normal exhalation by using a flexible tape without compressing skin and with 0.1 mm precision. [Waist circumference is attributed to the circumference at the novel and parallel to the crest of ilium measured with flexible tape]. Blood pressure was measured by a trained person and with standard methods. It was measured after 15 minutes relaxing in sitting position with right arm at heart level and supported by a firm rest. Mean of two measurements with 15 minutes interval was considered as the blood pressure of the subject. Subjects were emphasized not to consume tea, coffee, congestion iodine or to smoke cigarette 30 minutes prior to the blood pressure measurement.

In order to diagnose minor thalassemia MENTZER index, defined as ratio of MCV to RBC was used. In the case of MCV/RBC > 13, the diagnosis of minor thalassemia is confirmed [20]. Then, metabolic syndrome was calculated based on ATPIII criteria.

According to the current ATPIII criteria, the presence of 3 of the following 5 criteria is necessary to be considered as metabolic syndrome:

1. Abdominal obesity: waist circumference more than 102 cm in men and more than 88 cm in women.
2. TG ≥ 150 mg/dl or receiving medication for high triglyceride.
3. HDL cholesterol < 40 mg/dl in women or receiving medication for low HDL.
4. Blood pressure ≥ 130/85 mm/hg or receiving medicinal treatment for hypertension.
5. Fasting Plasma Glucose [FPG] ≥ 100 mg/dl or receiving medication for high FPG [21].

According to ATPIII criteria, hypertension is attributed to blood pressure ≥ 130/85 mm/hg, hypertriglyceridemia is attributed to TG ≥ 150 mg/dl, carbohydrate metabolism disorder is attributed to FBS ≥ 100 mg/dl, low HDL is attributed to HDL < 40 mg/dl in men and HDL < 50 mg/dl in women and abnormal waist circumference is attributed to waist circumference > 102 cm in men and > 85 cm in women.

Data analysis was performed through SPSS18 and using *t*-test and Chi-square for comparison of respectively quantitative and qualitative data. Significant statistical level was considered as p < 0.05. Statistical analysis of data were done by calculating mean, median, standard error, range of qualitative data and number and percent of quantitative data.

## Results

Table 1 presents a summary of demographic, anthropometric and clinical features of thalassemia and control groups. Mean age of thalassemia patients was 36.8 years and majority of them [76%] were female. As it is seen, the two groups show no significant difference in regard to age and sex. BMI and waist circumference were significantly lower in the thalassemia group compared to the control group. Thalassemia group in comparison to the control group had higher systolic blood pressure and FBS, while their LDL and HDL levels were lower. Based on ATP criteria, the prevalence of metabolic syndrome was lower in thalassemia group [12.7%] compared to the control group [36.7%] and according to the chi-square test this difference was statistically significant [p < 0.0001].

**Table 1:** **Demographic, anthropometric and clinical characteristics of two studied groups**

**Variable**	**Thalassemia group [n = 150]**	**Control group [n = 300]**	**p-value**
Age^1^ [year]	36.8 ± 7.9	36.6 ± 7.9	0.7
Sex^2^	36 [24]	70 [23.3]	0.8
Male			
Female	114 [76]	230 [76.7]	
BMI^1^ [kg/cm^2^]	25.1 ± 5	26.3 ± 4.7	0.01^*^
Waist circumference^1^ [cm]	79.1 ± 16.8	83.6 ± 11.1	<0.0001^*^
Systolic blood pressure^3^ [mm/hg]	110 [100-120]	110 [100-120]	0.01^*^
Diastolic blood pressure^3 ^[mm/hg]	70 [70-80]	70 [70-80]	0.08
FBS^3^ [mg/dl]	94 [84-107]	90 [82-100]	0.01^*^
Triglyceride^3^ [mg/dl]	108 [78.5-162.5]	113.5 [79.7-163]	0.8
Total Cholesterol^3^ [mg/dl]	164 [142-188.5]	189/5 [161-213.2]	<0.0001^*^

^1^Mean ± SD.

^2^Number [percent].

^3^Median [percentile 25, 75].

^*^statistically significant.

[Independent *t*-test, *Mann-Witney test].

From patients with metabolic syndrome in thalassemia group, 3 ones [8.3%] were male and 16 ones [14%] were female that shows no significant difference [p = 0.5].

Mean age of patients with metabolic syndrome in thalassemia group was 39.4 ± 8.5 years and in the control group it was 36.4 ± 7.8 years that shows no significant difference [p = 0.1]. In thalassemia group, mean BMI of patients with and without metabolic syndrome was respectively 31.3 ± 4.1 and 24.2 ± 4.4 that shows significant difference [p < 0.0001, Independent *t*-test].

Table 2 shows the prevalence of metabolic disorders and abnormal waist circumference in the two studied groups. As it is observed, the two groups show significant difference in regard to carbohydrates metabolism disorder. In thalassemia group, carbohydrate metabolism disorder had the highest prevalence [35.3%] and hypertriglyceridemia had the lowest prevalence [3.3%]. In the control group, Low HDL had the highest prevalence [80.5%] and hypertension had the lowest prevalence [11.1%].

**Table 2:** **Comparison of the two studied groups in regard to abdominal obesity and metabolic disorders [Chi-square]**

**Variable**	**Thalassemia group [%]**	**Control group [%]**	**p-value**
Abdominal obesity	30 [20.1]	78 [26.1]	0.1
Hypertension	13 [8.7]	33 [11.1]	0.5
Hypertriglyceridemia	5 [3.3]	85 [28.5]	<0.0001^*^
Low HDL	42 [28]	240 [80.5]	<0.0001^*^
Carbohydrates metabolism disorder	53 [35.3]	74 [24.8]	0.02^*^

^*^statistically significant.

In the thalassemia group, 13 ones [8.7%] and in the control group, 17 ones [5.7%] had diabetes [FBS ≥ 126 mg/dl], but this difference was not statistically significant [p = 0.2].

## Discussion

This case-control study showed that based on ATP criteria, the prevalence of metabolic syndrome in thalassemia patients is lower than that in normal population [16.7% vs. 36.7%].

In the present study, no significant difference was found between males and females with beta thalassemia in regard to developing metabolic syndrome. Indeed, in patients with minor beta thalassemia, no significant age difference was found between patients with metabolic syndrome and those without metabolic syndrome; even though, subjects with metabolic syndrome had higher BMI.

In the present study, in minor beta thalassemia group, carbohydrates metabolism disorder and in the control group, low HDL had the highest prevalence.

The prevalence of metabolic syndrome shows great variations among developing countries and has been reported from 13% in China to 30% in Iran [22].

In the national study for determining risk factors of non-communicable diseases performed in 2007 on 3027 Iranian persons aged 25-64 years living in 30 provinces, the prevalence of metabolic syndrome, based on ATPIII, was reported 34.7% with higher prevalence in females, urban residents and age group of 55-64 years [23].

In Tehran sugar and lipid study, the prevalence of metabolic syndrome was reported 33.7% and in both sexes it showed increase with age increase, though this increase was more in females [11]. In Fakhrzadeh et al. study on residents of one of the Tehran areas, the prevalence of metabolic syndrome was 29.9%. Moreover, sexual difference in relation to the prevalence of metabolic syndrome was seen. In the mentioned study, hypertriglyceridemia and hypertension were more frequent disorders seen in more than half of the population [24]. In another study performed on urban population of Zanjan/ Iran in 2009, the prevalence of metabolic syndrome was 23.7% with no difference in the two sexes and it showed increase with age increase. Moreover, low HDL showed the highest prevalence among other disorders [25]. In an epidemiologic cross-sectional study in Semnan province performed on 30-70 year old subjects on 2012, prevalence of metabolic syndrome, based on ATPIII criteria was 28.5% that showed an increase with age increase in both sexes, but it was more for females. Among risk factors of metabolic syndrome, based on ATPIII, high triglyceride had the highest prevalence [26].

In the present study, subjects with metabolic syndrome had significantly lower plasma levels of cholesterol and LDL and higher HDL level.

Hashemieh et al. [27] performed a study on lipid profile of 100 minor thalassemia patients and observed significant lower cholesterol and LDL levels in this group compared to normal subjects, but other factors [TG, HDL, VLD] showed no difference [27]. In Bordbar et al. study [28] performed on minor and major beta thalassemia patients in south Iran, lower triglyceride, cholesterol, LDL and HDL levels were seen in thalassemia patients compared to normal subjects [28].

In another study on major and minor thalassemia patients, performed by Haghpanah et al. [29], patient group and control group showed no difference in triglyceride level, while cholesterol and LDL levels of patients with major and minor thalassemia were lower compared to the control group and HDL level of minor thalassemia patients was lower than that of the control group [29].

In the present study, patient group had higher blood pressure and blood sugar compared to the control group, but diabetes and hypertension was not more prevalent in them. Further studies in regard to the prevalence of diabetes in thalassemia patients are recommended.

There was a limitation to this study. The study included only subjects who referred to Samen-ol-hojaj center for special disease. We suggest further studies with a sample size from community to confirm our findings.

## Conclusions

In general, metabolic syndrome has high prevalence in our country and is a health burden for our society. Therefore, more knowledge about this condition and factors affecting it seem to be necessary. The obtained results show lower prevalence of metabolic syndrome among minor thalassemia patients in comparison to the national statistics and propose minor thalassemia as a protective factor in metabolic syndrome. It is hoped that investigations on the mechanisms of this protective effect could suggest a solution for controlling this health problem.

One of the advantages of the present study is the fact that it has been performed for the first time in Iran.
